# A New Generation of FRET Sensors for Robust Measurement of Gα_i1_, Gα_i2_ and Gα_i3_ Activation Kinetics in Single Cells

**DOI:** 10.1371/journal.pone.0146789

**Published:** 2016-01-22

**Authors:** Jakobus van Unen, Anette D. Stumpf, Benedikt Schmid, Nathalie R. Reinhard, Peter L. Hordijk, Carsten Hoffmann, Theodorus W. J. Gadella, Joachim Goedhart

**Affiliations:** 1 Swammerdam Institute for Life Sciences, Section of Molecular Cytology, van Leeuwenhoek Centre for Advanced Microscopy, University of Amsterdam, P.O. Box 94215, NL-1090 GE, Amsterdam, The Netherlands; 2 Bio-Imaging-Center/Rudolf-Virchow-Zentrum and Department of Pharmacology and Toxicology, University of Wuerzburg, Versbacher Strasse 9, 97078, Wuerzburg, Germany; 3 Department of Molecular Cell Biology, Sanquin Research and Landsteiner Laboratory, Academic Medical Center, University of Amsterdam, NL-1066 CX, Amsterdam, the Netherlands; Boston University School of Medicine, UNITED STATES

## Abstract

G-protein coupled receptors (GPCRs) can activate a heterotrimeric G-protein complex with subsecond kinetics. Genetically encoded biosensors based on Förster resonance energy transfer (FRET) are ideally suited for the study of such fast signaling events in single living cells. Here we report on the construction and characterization of three FRET biosensors for the measurement of Gα_i1_, Gα_i2_ and Gα_i3_ activation. To enable quantitative long-term imaging of FRET biosensors with high dynamic range, fluorescent proteins with enhanced photophysical properties are required. Therefore, we use the currently brightest and most photostable CFP variant, mTurquoise2, as donor fused to Gα_i_ subunit, and cp173Venus fused to the Gγ_2_ subunit as acceptor. The Gα_i_ FRET biosensors constructs are expressed together with Gβ_1_ from a single plasmid, providing preferred relative expression levels with reduced variation in mammalian cells. The Gα_i_ FRET sensors showed a robust response to activation of endogenous or over-expressed alpha-2A-adrenergic receptors, which was inhibited by pertussis toxin. Moreover, we observed activation of the Gα_i_ FRET sensor in single cells upon stimulation of several GPCRs, including the LPA_2_, M_3_ and BK_2_ receptor. Furthermore, we show that the sensors are well suited to extract kinetic parameters from fast measurements in the millisecond time range. This new generation of FRET biosensors for Gα_i1_, Gα_i2_ and Gα_i3_ activation will be valuable for live-cell measurements that probe Gα_i_ activation.

## Introduction

The Gα_i_ subclass of heterotrimeric G-proteins consists of 3 members in humans, Gα_i1,2,3_ encoded by the genes GNAI1, GNAI2, GNAI3 [1] and is activated by a wide range of G-protein coupled receptors. The Gα_i_ family of G-proteins have been implicated in numerous pathologies, from involvement in obesity and diabetes [2], functions in the immune system [3] to their critical roles in several stages of cancer biology [4–7]. Activation of Gα_i_ is predominantly linked to the inhibition of adenylate cyclases, which leads to decreased cAMP accumulation in cells. However, activation of Gα_i_ has more recently been connected to several other molecular effectors, including PI3K/Akt [8,9], ERK [10] and c-Src [5].

The measurement of Gα_i_ activation is classically performed by measuring the inhibition of forskolin-induced cAMP production. Similar to phosphorylation assays further downstream, such measurements lack spatial resolution, have limited temporal resolution and can be influenced by considerable crosstalk and amplification or desensitization of the signal [11–13].

To investigate G-protein activation in a direct way with high spatiotemporal resolution, genetically encoded FRET (Förster Resonance Energy Transfer) or BRET (Bioluminescent Resonance Energy Transfer) biosensors can be employed [14]. These methods are based on the measurement of the non-radiative energy transfer from a donor molecule to an acceptor molecule, which only takes place when donor and acceptor are in close proximity of each other (<10nm). Changes in distance or orientation between the donor and acceptor dipole result in changes in the RET efficiency, which can be quantified.

The RET techniques allow for single cell recordings of the kinetics with millisecond resolution, which can be used to identify cell-to-cell heterogeneity and record pharmacokinetic parameters. Moreover, this approach has the potential to record GPCR activation under physiological conditions in vivo [15].

Gα_i_ has been successfully tagged at different internal sites with luciferase and used for BRET measurements between different Gα_i_ subunits and GPCRs [16–19] or Gγ [19]. FRET measurements between fluorescently tagged Gα_i1_, Gα_i2_ and Gα_i3_ and Gβ [20] or Gγ [21] have also been performed.

To perform FRET measurements, a spectrally overlapping donor and acceptor pair is necessary [24], and it was previously shown that the use of brighter fluorescent proteins can improve the sensitivity of FRET biosensor measurements [22,23]. In order to obtain robust FRET measurements that probe Gα_i_ activation, we have made fusions of Gα_i1_ Gα_i2_ and Gα_i3_ with the brightest and most photostable monomeric cyan fluorescent protein (CFP) currently available, mTurquoise2 (mTq2) [25]. As acceptor we have used circular permutated Venus (cpVenus) fused to Gγ_2_, which has previously been used as acceptor in a single plasmid Gα_q_ FRET sensor [26]. We use a single plasmid strategy to facilitate transfection protocols and allow a well-defined donor and acceptor expression ratio in cells [27]. This expression strategy should greatly facilitate the use and reproducibility of the results of these sensors. We present the construction strategy, validation and characterization of this new generation of FRET sensors for the activation of Gα_i1_ Gα_i2_ and Gα_i3_. These biosensors are very well suited for live cell microscopy and can be used for fast kinetic measurements in the millisecond range, allowing pharmacological drug characterization and determination of on- and off-kinetics for agonists and antagonists at Gα_i_–coupled GPCRs.

## Results

### Generation of constructs

**Fig 1:**
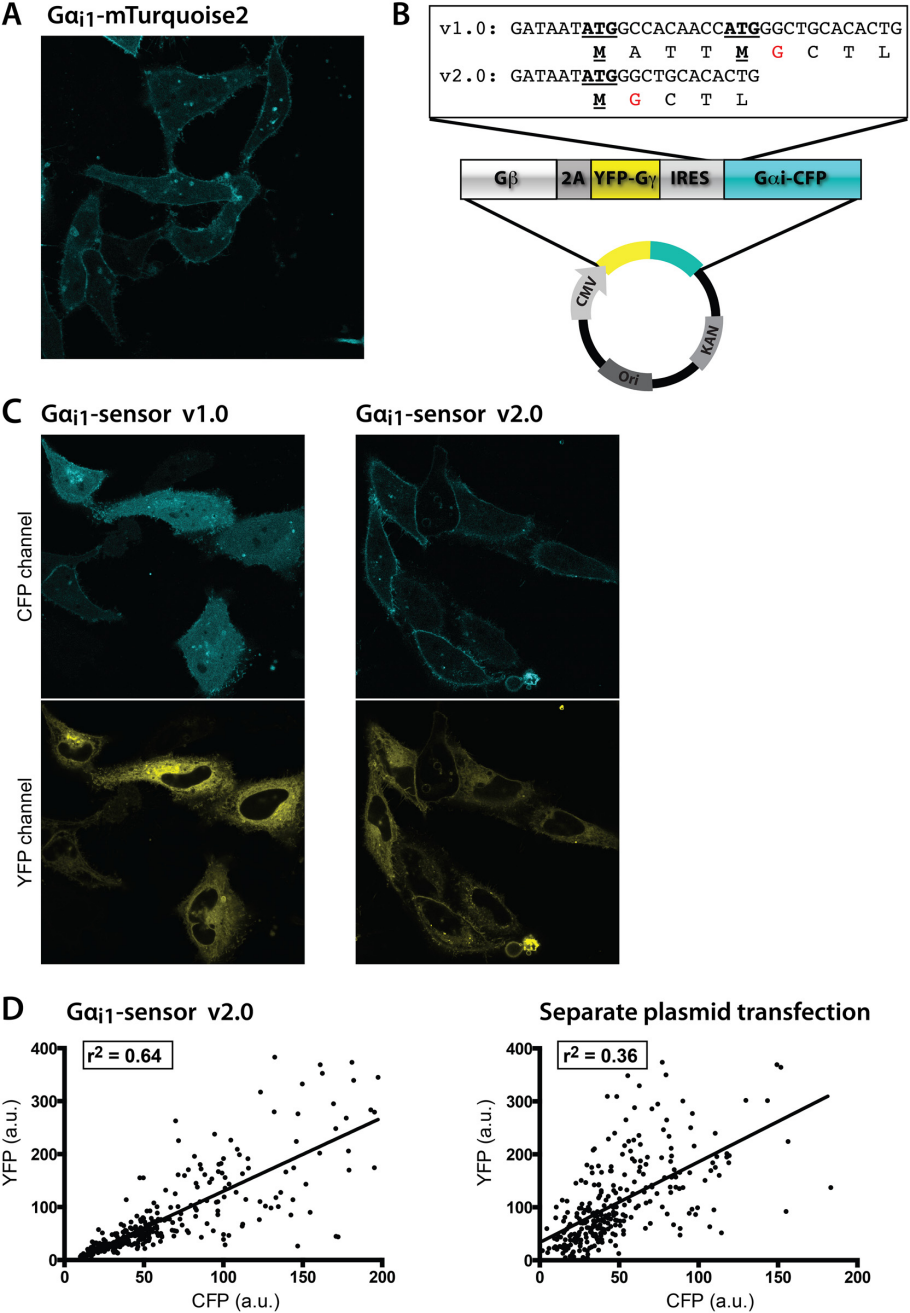
Development and characterization of the new Gα_i1_-sensor. (A) Representative image showing the plasma membrane localization of Gα_i1_ fused to mTurquoise2-Δ9, expressed in HeLa cells. (B) Schematic overview of the plasmid containing pGβ-2A-YFP-Gγ_2_-IRES-Gα_i1_-CFP, driven by a CMV promoter. The inset shows the DNA sequence encoding the end of the IRES sequence and the start of the Gα_i1_ sequence. The proposed protein translation is shown in the line below the DNA sequence (single letter abbreviations of the amino acids). (C) Confocal images of the localization of Gα_i1_-mTurquoise2-Δ9 (*top row*) and cp173Venus-Gγ_2_ (*bottom row*) in HeLa cells, for variant 1.0 (*left column*) and variant 2.0 (*right column*) of the Gα_i1_-sensor. (D) Quantitative co-expression analysis of the CFP and YFP channels of the cp173Venus-Gγ_2_ and Gα_i1_-mTurquoise2-Δ9 transfections in HeLa cells. Single plasmid transfection (*left*) versus the transfection of the separate plasmids (*right*). The dots depict the CFP and YFP intensity, quantified from individual single cells. The r^2^ is the coefficient of determination. Width of the individual images in A and C is 143μm.

The monomeric CFP variant mTurquoise2, the preferred donor in CFP-YFP FRET pairs due to its high quantum yield and photostability [25], was inserted into Gα_i1_ after the alanine on position 121 in the αB-αC loop. This insertion site that was previously shown to retain nucleotide exchange and GTPase reaction rates comparable to wild-type protein [20]. Gα_i1_-mTq2 displays plasma membrane localization when expressed in HeLa cells (Fig 1A). Trial experiments were performed to examine whether Gα_i1_-mTq2 is suitable for measuring, by means of FRET, the activation of the heterotrimeric G-protein complex upon GPCR activation. To this end, both Gβ as well as Gγ can be tagged with an acceptor to measure heterotrimeric G-protein activation by FRET [20,21]. We have previously shown that the highest FRET contrast, for Gα_q_, is obtained with cpVenus-Gγ_2_ [26]. Since Gα_i_ has high structural homology to Gα_q_ and the site at which mTurquoise2 is inserted is similar, we decided to employ the same FRET acceptor here. Hence, to introduce a labeled heterotrimeric G-protein complex in cells, we co-expressed Gα_i1_-mTq2 together with the FRET acceptor cpVenus-Gγ_2_ and untagged Gβ_1_ [26]. Stimulation of the co-expressed α_2_ adrenergic receptor (α_2_AR) with UK14,304 shows a rapid increase in CFP fluorescence and a concomitant loss of sensitized emission from the YFP channel, reflecting a loss of FRET. The loss of FRET can be interpreted as a dissociation of the heterotrimer or a change in relative conformation of donor and acceptor. To enable robust co-expression of the different components of the multimeric FRET sensor, we introduced the subunits on the same plasmid, as we reported previously for a Gα_q_ sensor (Fig 1B) [26]. This strategy uses a viral 2A peptide and an IRES sequence to ensure optimal relative levels of the donor (Gα_i1_-mTq2) and acceptor (cpVenus-Gγ_2_) within single cells, while minimizing cell-to-cell expression heterogeneity in the sample. After generating our first variant, we noticed that Gα_i1_-mTq2 was mislocalised in the cytoplasm in many cells (Fig 1C). The Gα_i1_ subunit is myristoylated and requires a glycine residue immediately following its starting methionine. Detailed inspection of the plasmid sequence revealed an additional starting codon for the Gα_i1_-mTq2 upstream of the native start-codon, generated by the IRES sequence (Fig 1B). We hypothesized that in our first variant of the sensor, most of the Gα_i1_-mTq2 protein produced was translated from the upstream methionine in the IRES sequence, which does not result in myristoylated protein. We removed the upstream methionine by whole-vector PCR (see material and methods), which only leaves the native starting codon of Gα_i1_-mTq2, followed by a Glycine providing the consensus sequence for myristoylation. Indeed, after transfecting cells with the new plasmid, we observed correctly localized Gα_i1_-mTq2 (Fig 1B and 1C). A Gα_i2_-sensor and Gα_i3_-sensor were constructed in a similar way. To examine the co-expression of the three subunits (Gα_i1_-mTq2, Gβ_1_ and cpVenus-Gγ_2_) from a single plasmid versus three separate plasmids, we quantified the CFP and YFP fluorescence in these two experimental conditions (Fig 1D). The CFP and YFP fluorescence in the single plasmid strategy transfection had a coefficient of determination r^2^ of 0.64, whereas transfections with the three separate plasmids showed a coefficient of determination r^2^ of 0.36 between CFP intensity and YFP intensity. In other words, the correlation between CFP and YFP expression is better in the single plasmid configuration, indicating a clear advantage of this design. An additional advantage of this plasmid is the 3:1 protein expression upstream and downstream of the IRES sequence, which has previously been shown to result in a preferred donor (CFP) and acceptor (YFP) expression ratio for an analogous Gα_q_ FRET sensor [27]. Finally, the single plasmid constructs will simplify introduction into primary cells, the generation of stable cell lines or transgenic organisms with Gα_i_-sensors.

### Performance in GPCR activation assays

**Fig 2:**
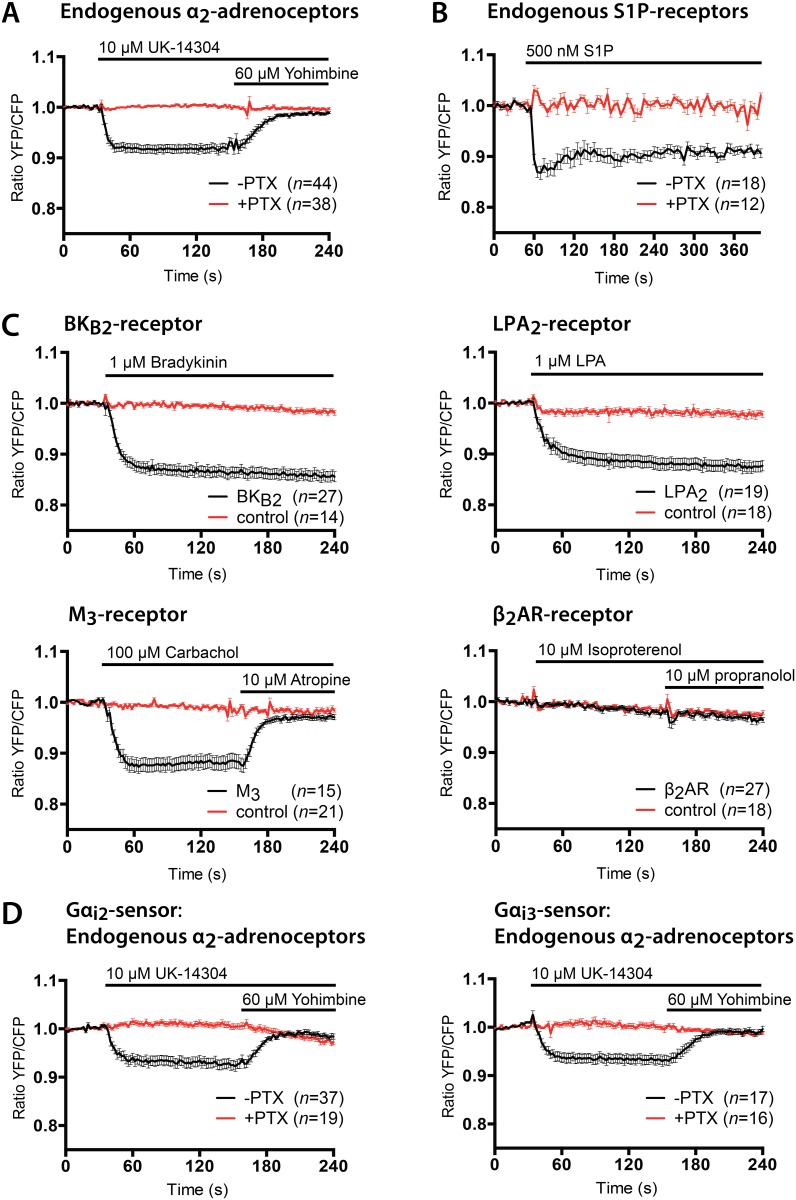
Performance of the Gα_i_-sensors in single cell GPCR signaling assays. (A) FRET ratio-imaging experiments in HeLa cells transfected with the Gα_i1_-sensor. Rapid loss of FRET, observed by a decreased YFP/CFP ratio, after stimulation of the cells with 10μM UK-14,304, an α_2_AR specific agonist, addition of 60μM Yohimbine returns the FRET ratio towards baseline levels. Overnight treatment with (100ng/mL) PTX abolishes the response on the Gα_i1_-sensor in UK-14304 stimulated cells. (B) FRET ratio-imaging experiments in Huvecs transfected with the Gα_i1_-sensor. A sustained loss of FRET is observed after stimulation with 500nM S1P (Sphingosine-1-phosphate). Overnight treatment with (100ng/mL) PTX abolishes the response on the Gα_i1_-sensor in S1P stimulated cells. (C) FRET ratio-imaging experiments of HeLa cells transfected with the Gα_i1_-sensor and BK_2B_ (*top-right*), LPA_2_ (*top-left*), M_3_ (*bottom-right*) and β_2_AR-2A2-mCherry (*bottom-left*) were stimulated with 1μM bradykinin (BK_2B_), 1μM lysophosphatidic acid (LPA_2_), 100μM carbachol and 10μM atropine (M_3_) or 10μM isoproterenol and 10μM propranolol (β_2_AR). HeLa cells transfected with BK_2B_, LPA_2_ and M_3_ receptors show a clear change in YFP/CFP FRET ratio upon addition of their respective agonists, whereas stimulation of the β_2_AR does not alter the FRET ratio of the Gα_i1_-sensor. In the control conditions, HeLa cells with only the Gα_i1_-sensor transfected received identical stimulations. (D) FRET ratio-imaging experiments in HeLa cells transfected with the Gα_i2_-sensor or Gα_i3_-sensor. Rapid loss of FRET is observed after stimulation of the cells with 10μM UK-14,304, subsequent addition of 60μM Yohimbine returns the FRET ratio towards baseline levels. Overnight treatment with (100ng/mL) *pertussis toxin* (PTX) abolishes the response on the Gα_i2_ and Gα_i3_ biosensors in UK-14304 stimulated cells. HeLa cells were stimulated with an agonist at *t* = 32s and an antagonist was added at *t* = 152s where indicated. Huvec cells were stimulated with S1P at *t* = 55s. Time traces show the average ratio change of YFP/CFP fluorescence (±s.e.m). Average curves consist of data from at least 3 independent experiments, conducted on different days, with the indicated number of cells (*n*) per condition.

To test the new Gα_i1_ biosensor in live cell imaging, we employed a well-characterized GPCR known to couple to Gα_i1_, the α_2_ adrenergic receptor (α_2_AR). HeLa cells, shown before to contain the α_2_AR endogenously [20], were transfected with the Gα_i1_ biosensor. Upon addition of 10μM UK14,304 we observed a robust loss of FRET by measuring the ratio between the YFP and CFP fluorescence of the Gα_i1_ biosensor, which was reversed back to baseline by the addition of 60μM of the α_2_AR antagonist Yohimbine (Fig 2A). *Pertussis toxin* (PTX) has been shown to inactivate Gα_i_ signaling in cells via ADP-ribosylation of the Gα_i_ subunit [28], which prevents its interaction with GPCRs. The activation of the Gα_i1_ was completely abolished by overnight incubation with PTX, showing that Gα_i1_-mTq2 protein fusion is still PTX-sensitive (Fig 2A). To confirm that the sensor can be used to assay Gα_i1_ activation of endogenous receptors in primary cells, we repeated this experiment in HUVEC (human umbilical vein endothelial cells). Addition of a well-known stimulant for HUVECs, S1P [29], caused a sustained decrease in FRET ratio of the Gα_i1_-sensor (Fig 2B), overnight treatment with PTX completely abolished this response. Next, to investigate how robust the Gα_i1_ sensor performs on other GPCR activation assays, we tested a variety of GPCRs shown to couple to Gα_i_. The bradykinin 2B (BK_2B_) receptor [30], lysophosphatidic acid 2 (LPA_2_) receptor [31,32] and muscarinic acetylcholine 3 (M_3_) receptor [33,34] were co-transfected with the Gα_i1_ biosensor in HeLa cells. Upon stimulation with the relevant agonists, all three receptors showed a sustained decrease in FRET ratio of the Gα_i1_ biosensor (Fig 2C). The M_3_ receptor also showed a full recovery back to baseline of the FRET ratio after addition of the antagonist atropine. The M_3_ receptor is mainly known for its signaling via Gα_q_. Still, previous studies have shown Gα_i_ activation via the M_3_ receptor [19,33–36], fitting with with our observations.

In the control conditions, e.g. absence of over-expressed GPCR, we stimulated HeLa cells with the relevant agonist and antagonist, and we observed only a very minor response on the Gα_i1_ sensor in the case of LPA stimulation. This is most likely due to the activation of endogenous LPA receptors in HeLa cells [37]. When we co-transfected the β_2_ adrenergic receptor (β_2_AR), none of the cells showed Gα_i1_ activation in response to the agonist and antagonist treatment (Fig 2C). Of note, β_2_AR is a classical activator of Gα_s_ but switching to Gα_i_ has been reported under certain conditions [38]. Our results fit with the only study that we are aware of that uses similar tools (BRET based sensors) and similar conditions (over-expressed β_2_AR and heterotrimeric G-protein sensors) [19]. Also in that case no activation of Gα_i1_ was observed by β_2_AR stimulation (and only little activation of Gα_i2_ and Gα_i3_, which was >10-fold lower than activation by the alpha-2C adrenergic receptor, a strong activator of Gα_i_).

To verify the performance of the Gα_i2_ and Gα_i3_ biosensors, we transfected HeLa cells with their respective plasmids. Similar to Gα_i1_ biosensor experiment in Fig 2A we observed a robust loss of FRET after addition of 10μM UK14,304, and the signal returned to baseline upon addition of 60μM Yohimbine (Fig 2D). Under these experimental conditions we did not observe substantial differences in the activation kinetics or amplitude of the responses between the three different Gα_i_ subunits. Both Gα_i2_-mTq2 and Gα_i3_-mTq2 are still sensitive to PTX treatment, as shown by the abolishment of the FRET response after overnight incubation with PTX (Fig 2D).

### Fast kinetic measurements

**Fig 3:**
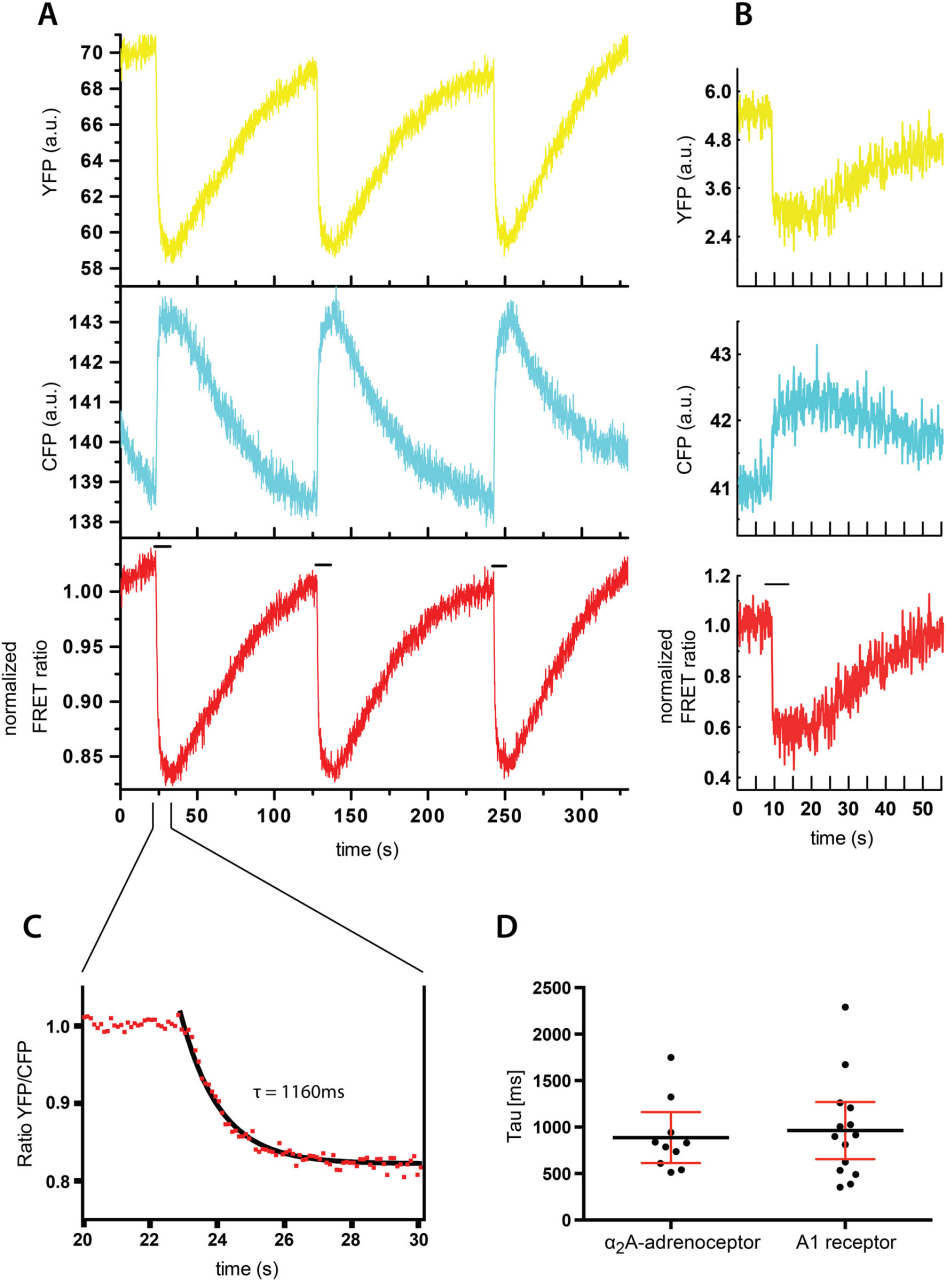
Performance of the Gα_i1_-sensor in kinetic measurements. (A) HEK293 cells transfected with the Gα_i1_-sensor and the α_2_AR were repeatedly stimulated with 20μM norepinephrine during intervals that are indicated by short horizontal lines. The presented data is representative for at least six different transfections performed on six experimental days. Top panel: YFP emission, center panel: CFP emission, bottom panel: corrected and normalized FRET ratio. (B) HEK293 cells transfected with the Gα_i1_-sensor and the Adenosine A1-receptor were stimulated with 30μM adenosine, indicated by the short horizontal line. The presented data is representative for at least six different transfections performed on six experimental days. Top panel: YFP emission, center panel: CFP emission, bottom panel: corrected and normalized FRET ratio. (C) A close-up of the on-kinetics of Gα_i1_ activation, showing the normalized FRET ratio during the first stimulation of the experiment in (A), fitted to a one component exponential decay function with tau = 1160ms and amplitude = 0.18 (R = 0.99). (D) Scatter plot showing the average exponential time constants (tau) of pooled data from (n = 10) individual fits of HEK293 cells transfected with the Gα_i1_-sensor and the α_2_AR stimulated with 100μM norepinephrine or pooled data (n = 14) from individual fits of the Gα_i1_-sensor and the Adenosine A1-receptor stimulated with 30μM adenosine, respectively. Error bars indicate 95% CI.

In order to look at the sub-second kinetics of Gα_i1_ activation in living cells in more detail, HEK293 cells were co-transfected with the Gα_i1_-sensor and the α_2_AR or adenosine A1 receptor, respectively. Using a fast perfusion system for ligand application, single-cell FRET measurements show a rapid loss in FRET ratio of more than 15% after short-term application of 20μM norepinephrine. After ligand washout, the FRET signal returns to baseline levels. This could be reproduced several times without any apparent loss in signal amplitude (Fig 3A). A similar response was observed for the adenosine A1 receptor after short application of the endogenous ligand adenosine (30μM) (Fig 3B).

These fast FRET measurements can be used to estimate the on-kinetics of Gα_i1_ activation with sub second resolution (Fig 3C), as shown by a close-up of the first stimulation in the experiment shown in Fig 3A. The curve was fitted to a one component exponential decay function as previously described [39], resulting in an exponential time constant (τ) of 1160ms.

To assess the precise on-rate kinetics of the Gα_i1_-sensor, cells were stimulated with saturating ligand concentrations (100μM norepinephrine or 30μM adenosine). Each individual response was fitted to a one component exponential, this resulted in average τ values for α_2_AR of 887ms and for adenosine A1 of 963ms (Fig 3D), corresponding to half-times of 614ms and 668ms respectively. These values are in good agreement with earlier observations for G-protein activation by FRET [21,40,41].

## Concluding Remarks

In this manuscript we describe the design, construction and characterization of three new FRET biosensors for the measurement of Gα_i1_, Gα_i2_, Gα_i3_ activation. The new sensors contain a Gα subunit fused to the donor fluorophore, mTurquoise2, and the Gγ subunit fused to cp173Venus, as it was previously shown that this combination for a Gα_q_ FRET sensor provides the largest dynamic range [26]. The three subunits of the heterotrimer (Gα_i_-mTq2, Gβ_1_ and cpVenus-Gγ_2_) were configured on a single plasmid, enabling robust co-expression with a preferred stoichiometry. We show that these sensors are well suited for live cell microscopy and extracting kinetic parameters by single-cell ratiometric FRET imaging. The standardized layout of these FRET biosensors for G-protein activation will improve reliability and reproducibility of experiments within and between laboratories. This is exemplified in this paper by the robust performance of the Gα_i1_ sensor in three different laboratories, without optimization of the experimental conditions.

One limitation of energy transfer based biosensors for heterotrimeric G-proteins is that they depend on overexpression of the heterotrimer, which may affect the natural preference of the GPCR for a certain class of heterotrimeric G-proteins. Tagging the endogenous subunits with fluorescent proteins can potentially alleviate this.

The exquisite sensitivity of these sensors enables the robust detection of Gα_i_ activation in primary cells via endogenous GPCRs. Moreover, these biosensors can be used to directly compare the preferential activation patterns of Gα_i1_ Gα_i2_ and Gα_i3_ between different Gα_i_ coupled GPCRs, which can aid the development of therapeutic strategies targeting Gα_i_ signaling pathways [42].

## Methods

### Construction of fluorescent protein fusions

To insert mTurquoise2-Δ9 (abbreviated here as mTq2) [25] in the Gα_i1_, Gα_i2_ and Gα_i3_ proteins, a version of mTq2 with Age1 restriction sites at its N-terminal and C-terminal was constructed by amplifying mTurquoise2 with forward primer 5’-ATaccggttctATGGTGAGCAAGGGCG-3’ and reverse primer 5’-TAaccggtGATCCCGGCGGC-3’. To introduce an Age1-site in the Gα_i1_-citrine, we performed a whole-vector PCR on template RnGalpha_i1_-Citrine [20] with forward primer 5’-ATaccggtGAACTCGCCGGCGTCATA-3’ and reverse primer 5’-ATaccggtCGCGGTCATAAAGCCTTC-3’. To introduce an Age1-site in the Gα_i2_-citrine, we performed a whole-vector PCR on template HsGalpha_i2_-Citrine [20] with forward primer 5’-ATaccggtGAGGAGCAAGGCGTGCT-3’ and reverse primer 5’- TAaccggtGGCGGTGCAGGACAGT-3’. The cDNA containing the coding sequence for HsGalpha_i3_ with an Age1 site was synthesized by Eurofins (www.eurofins.nl). Cutting the mTq2 PCR product and the new Gα_i1_, Gα_i2_ and Gα_i3_ vectors with Age1 and subsequent ligation resulted in RnGalpha_i1_ tagged with mTq2 after position 121, and HsGalpha_i2,3_ tagged with mTq2 after position 114, analogous to a previously reported functionally tagged Gα_i1,2,3_ [20].

To construct variant 1.0 of the Gα_i1_ sensor, a PCR was performed on the mTq2-Gα_i1_ plasmid, with forward primer 5’-AGGTCTATATAAGCAGAGC-3’ and reverse primer 5’-TATggatccAGCTTAGAAGAGACCACAGTC-3’ to introduce a BamHI site at the C-terminus and an NcoI site at the N-terminus. Next a triple ligation was performed with the PCR product (cut with BamHI and NcoI), a vector containing pGβ_1_-T2A-cp173Venus-Gγ_2_ [43](cut with BamHI and SacII), and a vector containing pPRIG-IRES [44] (cut with NcoI and SacII). The resulting plasmid, pGβ_1_-2A-YFP-Gγ_2_-IRES-MATT-Gα_i1_-CFP, co-expresses MATT-Gα_i1_-mTurquoise2-Δ9 (impaired plasma membrane localization), pGβ_1_ and cp173Venus-Gγ_2_ (Fig 1B).

To construct variant 2.0 of the Gα_i1_-sensor, we performed a mutagenesis PCR with variant 1.0 as template, by amplifying with forward 5'-GAAAAACACGATGATAATATGGGCTGCACACTGAGC-'3 and 5'-GCTCAGTGTGCAGCCCATATTATCGTGTTTTTC-3'. The resulting plasmid, pGβ_1_-2A-cp173Venus-Gγ_2_-IRES-Gα_i1_-mTurquoise2-Δ9, co-expresses Gα_i1_-mTq2 (properly located at the plasma membrane), pGβ_1_ and cpVenus-Gγ_2_ (Fig 1B).

To create a single plasmid sensor for Gα_i2_ and Gα_i3_, we performed an overlap extension PCR [45]. Gα_i2_-mTq2 was amplified with forward primer ‘5-acgatgataatATGGGCTGCACCGTGA-3’ and reverse primer ‘5 -TATtctagaAGCTCAGAAGAGGCCGCAGT-3’, and Gα_i3_-mTq2 was amplified with forward primer ‘5-acgatgataatATGGGCTGCACGTTGA-3’ and reverse primer ‘5 -TATtctagaAGCTTAATAAAGTCCACATTCCT-3’. Another PCR was performed on the previously described [26] single plasmid Gα_q_-sensor, with forward primer ‘5- GAAGTTTTTCTGTGCCATCC -3’ and reverse primer ‘5—GCAGCCCATattatcatcgtgtttttcaaag -3’. Subsequently, the above described PCR product of Gα_i2_-mTq2 or Gα_i3_-mTq2 were mixed with the PCR product of the Gα_q_-sensor and used as template for a third PCR with forward primer ‘5- GAAGTTTTTCTGTGCCATCC-3’ and reverse primer ‘5- TATtctagaAGCTCAGAAGAGGCCGCAGT-3’, and forward primer ‘5- GAAGTTTTTCTGTGCCATCC-3’ and reverse primer ‘5- TATtctagaAGCTTAATAAAGTCCACATTCCT-3’, respectively. The resulting PCR products were then ligated into the Gα_q_-sensor backbone with SacII and XbaI, resulting in pGβ-2A- cp173Venus -Gγ_2_-IRES-Gα_i2_-mTurquoise2-Δ9 and pGβ_1_-2A- cp173Venus-Gγ2-IRES-Gα_i3_-mTurquoise2-Δ9, respectively. The sequences of the plasmids are available upon request. The plasmids will be distributed through Addgene: http://www.addgene.org/Dorus_Gadella/. RnGα_i1_-mCitrine and HsGα_i2_-mCitrine were a kind gift from Scott Gibson [20]. Note that RnGα_i1_ coding sequence differs only one amino acid from human Gα_i1_ (S98A). The LPA_2_ receptor was obtained from cDNA.org. BK_2_R [46], α_2_AR [21], M_3_R [47] and the A1 receptor [48] were previously described. β_2_AR-P2A-mCherry was a kind gift from Anna Pietraszewska (University of Amsterdam).

### Cell culture and sample preparation

HeLa cells (American Tissue Culture Collection: Manassas, VA, USA) were cultured at the University of Amsterdam (Amsterdam, the Netherlands) using Dulbecco’s Modified Eagle Medium (DMEM) supplied with Glutamax, 10% FBS, Penicillin (100 U/ml) and Streptomycin (100μg/ml). All cell culture media were obtained from Invitrogen (Bleiswijk, NL).

Cells were transfected in a 35 mm dish holding a glass 24 mm Ø #1 coverslip (Menzel- Gläser, Braunschweig, Germany), using 1–2μl Lipofectamine 2000 according to the manufacturer’s protocol (Invitrogen), 0.5–1μg plasmid cDNA and 50μl OptiMeM (Life Technologies, Bleiswijk, NL). After overnight incubation at 37°C and 5% CO_2_, coverslips were mounted in an Attofluor cell chamber (Invitrogen, Breda, NL) and submerged in microscopy medium (20mM HEPES (PH = 7.4), 137 mM NaCL, 5.4mM KCL, 1.8 mM CaCL_2_, 0.8mM MgCl_2_ and 20mM glucose). All live cell microscopy was done at 37°C.

Human umbilical vein endothelial cells (HUVECs) were purchased from Lonza and cultured at Sanquin Blood Supply (Amsterdam, the Netherlands) on FN-coated dishes in EGM-2 medium, supplemented with singlequots (Lonza, Verviers, Belgium). HUVECs were used at passage number 4 or 5. The Neon transfection system (MPK5000, Invitrogen) and a corresponding Neon transfection kit (Invitrogen) were used as transfection method. A single pulse was generated at 1300 Volt for 30ms to microporate HUVECs with 2μg cDNA, cells were subsequently seeded on FN-coated glass coverslips.

For the rapid kinetic measurements of Gα_i1_ activation, HEK293 cells were cultivated at the University of Wuerzburg (Wuerzburg, Germany) in Dulbecco’s modified Eagle’s medium (DMEM) supplemented with 10% fetal calf serum, L-Glutamine (2 mM) (PAN Biotech GmbH, Aidenbach, Germany), Penicillin (100 U/ml), and Streptomycin (100 μg/ml) and kept at 37°C in a 7% CO2 atmosphere. Cells were harvested and seeded onto D-Polylysine-coated 24 mm glass coverslips at ~40% confluency. After three hours cells were transiently transfected with 1.0μg of receptor (α_2_AR or adenosine A1) and 3.0 μg pGβ_1_-2A-YFP-Gγ_2_-IRES-Gα_i1_-mTq2 cDNA per 6-well plate using Effectene^®^ transfection reagent (Qiagen), according to the manufacturer’s protocol. Growth medium was renewed after 24h and measurements were performed after a total incubation time of 48h. The cells were kept in microscopy medium (140 mM NaCl, 5.4 mM KCl, 2 mM CaCl2, 1 mM MgCl2, 10 mM HEPES, pH 7.3) and permanently superfused with this buffer or buffer supplemented with the appropriate ligand, using a computer-assisted solenoid valve-controlled rapid superfusion device (ValveLink 8.2, Automate Scientific).

### Widefield microscopy

Ratiometric FRET measurements in HeLa cells (results presented in Fig 2A, 2C and 2D) were performed using a wide-field fluorescence microscope (Axiovert 200 M; Carl Zeiss GmbH, Germany) at the University of Amsterdam (Amsterdam, the Netherlands), kept at 37°C, equipped with an oil-immersion objective (Plan-Neo- fluor 40×/1.30; Carl Zeiss GmbH) and a xenon arc lamp with monochromator (Cairn Research, Faversham, Kent, UK). Images were recorded with a cooled charged-coupled device camera (Coolsnap HQ, Roper Scientific, Tucson, AZ, USA). Typical exposure times ranged from 75ms to 150ms, and camera binning was set to 4x4. Fluorophores were excited with 420 nm light (slit width 30nm) and reflected onto the sample by a 455DCLP dichroic mirror and CFP emission was detected with a BP470/30 filter, and YFP emission was detected with a BP535/30 filter by rotating the filter wheel. In the co-expression experiments, YFP was excited with 500nm light (slit width 30nm) and reflected onto the sample by a 515DCXR dichroic and emission was detected with a BP535/30 filter. Acquisitions were corrected for background signal and, for FRET ratio imaging, bleedthrough of CFP emission in the YFP channel (55% of the intensity measured in the CFP channel).

For the FRET experiments in HUVECs (results presented in Fig 2B), a Zeiss Observer Z1 microscope was used at Sanquin Blood Supply (Amsterdam, the Netherlands) with a 40x NA 1.3 oil immersion objective and an HXP 120 V excitation light source. CFP was excited through a FRET filter cube (Exciter ET 436/20x, and 455 DCLP dichroic mirror (Chroma, Bellows Falls, Vermont, USA)). The emission was directed to an attached dual camera adaptor (Carl Zeiss GmbH, Germany) controlling a 510 DCSP dichroic mirror (Chroma, Bellows Falls, Vermont, USA). Emission wavelengths between 455–510 nm are directed to an emission filter ET 480/40 (Chroma, Bellows Falls, Vermont, USA) and then captured by a Hamamatsu ORCA-R2 camera. Emission wavelength 510 nm and higher are directed to an ET 540/40m emission filter (Ludl Electronic Products, NY, USA) and then captured by a second Hamamatsu ORCA-R2 camera. Image acquisition was performed using Zeiss-Zen 2011 microscope software. All acquisitions were corrected for background signal. Acquisitions were corrected for background signal and bleedthrough of CFP emission in the YFP channel (62% of the intensity measured in the CFP channel).

For the rapid kinetic measurements of Gα_i1_ activation (results presented in Fig 3), imaging was performed on a Zeiss Axiovert 200 inverted microscope at the University of Wuerzburg (Wuerzburg, Germany), equipped with an oil immersion 63x objective lens and a dual-emission photometric system (Till Photonics) as described before [21]. The transfected cells were excited with light from a polychrome IV (Till Photonics). Illumination was set to 40ms out of a total integration time of 100ms. CFP (480 ± 20 nm), YFP (535 ± 15 nm), and FRET ratio (YFP/CFP) signals were recorded simultaneously (beam splitter DCLP 505 nm) upon excitation at 436 ± 10 nm (beam splitter DCLP 460 nm). Fluorescence signals were detected by photodiodes and digitalized using an analogue-digital converter (Digidata 1440A, Axon Instruments). All data were recorded on a PC running Clampex 10.3 software (Axon Instruments). Resulting individual traces were fit to a one component exponential decay function to extract the exponential time constant, tau [39]. The halftime of activation (t_1/2_) is defined as τ*ln2. In dynamic experiments, cells were stimulated with UK14,304 (10μM), Yohimbine (60μM), Bradykinin (1μM), LPA (1μM), Carbachol (100μM), Atropine (10μM), Isoproterenol (10μM), Propranolol (10μM), S1P (500nM), 20μM or 100μM norepinephrine or 30μM adenosine at the indicated time points. ImageJ (National Institute of Health) was used to analyze the raw microscopy images. Further processing of the data was done in Excel (Microsoft Office) and graphs and statistics were conducted using Graphpad version 6.0 for Mac, GraphPad Software, La Jolla California USA, www.graphpad.com.

### Confocal microscopy

HeLa cells transfected with the indicated constructs were imaged using a Nikon A1 confocal microscope equipped with a 60x oil immersion objective (Plan Apochromat VC, NA 1.4). The pinhole size was set to 1 Airy unit (<0.8μm).

Samples were sequentially excited with a 457nm and a 514nm laser line, and reflected onto the sample by a 457/514 dichroic mirror. CFP emission was filtered through a BP482/35 emission filter; YFP emission was filtered through a BP540/30 emission filter. To avoid bleed-through, images were acquired with sequential line scanning modus. All acquisitions were corrected for background signal.

## Supporting Information

S1 Data The compressed file contains all the data that was used in this manuscript. (ZIP)
