# Supplementary figures and images for: A New Generation of FRET Sensors for Robust Measurement of Gαi1, Gαi2 and Gαi3 Activation Kinetics in Single Cells
Source: PLoS One. 2016 Jan 22;11(1):e0146789. doi: 10.1371/journal.pone.0146789 (PMC4723041; doi:10.1371/journal.pone.0146789)

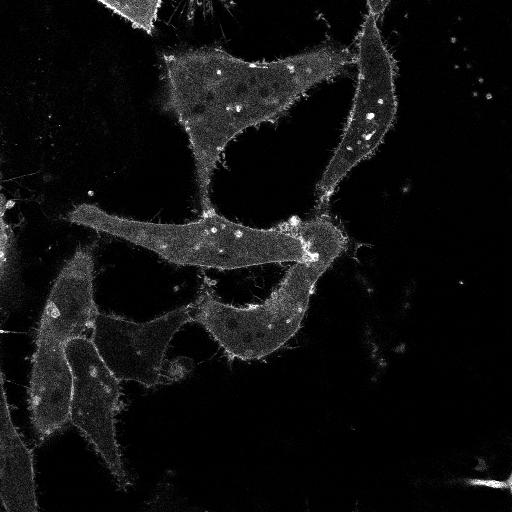

Supplement: S1 Data — (ZIP) [file pone.0146789.s001.zip › vanUnen_Gai-Data_PLoSOne/Figure_01/Galphai1_mTurqoise2.tif]

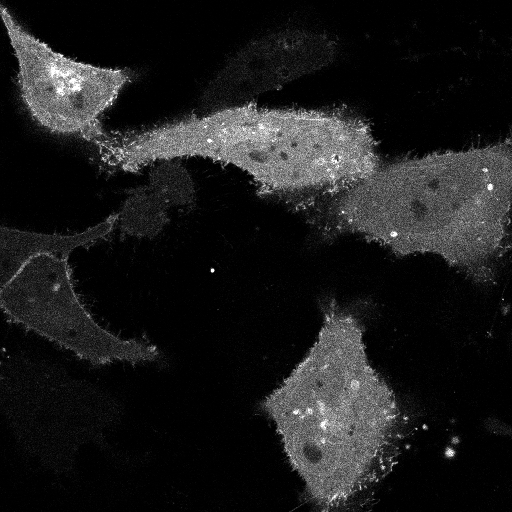

Supplement: S1 Data — (ZIP) [file pone.0146789.s001.zip › vanUnen_Gai-Data_PLoSOne/Figure_01/Galphai1_sensor_v1.0_CFP-channel.tif]

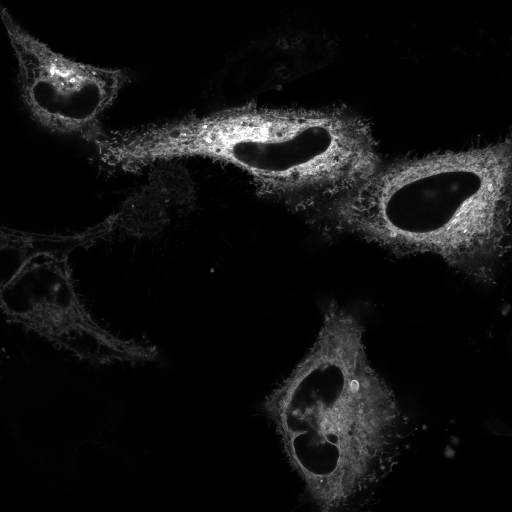

Supplement: S1 Data — (ZIP) [file pone.0146789.s001.zip › vanUnen_Gai-Data_PLoSOne/Figure_01/Galphai1_sensor_v1.0_YFP-channel.tif]

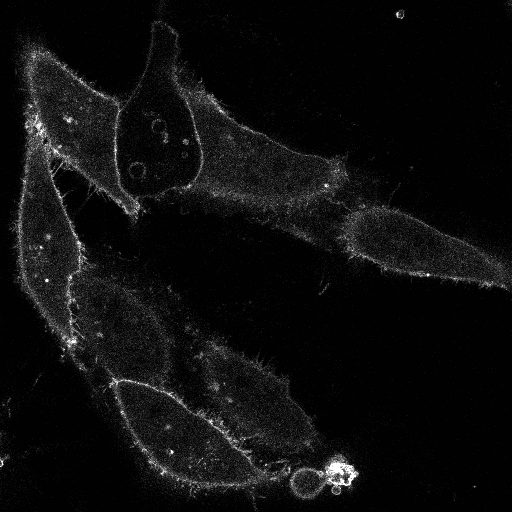

Supplement: S1 Data — (ZIP) [file pone.0146789.s001.zip › vanUnen_Gai-Data_PLoSOne/Figure_01/Galphai1_sensor_v2.0_CFP-channel.tif]

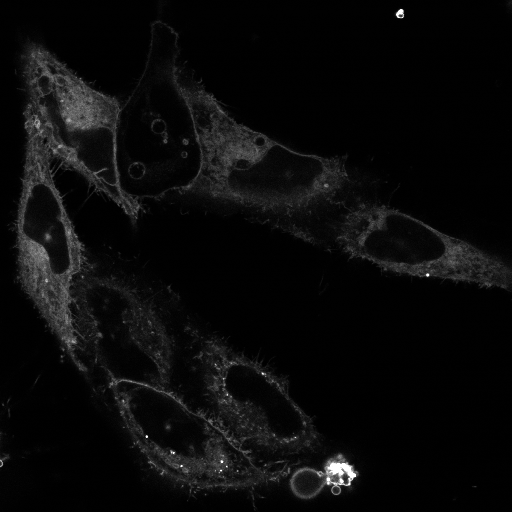

Supplement: S1 Data — (ZIP) [file pone.0146789.s001.zip › vanUnen_Gai-Data_PLoSOne/Figure_01/Galphai1_sensor_v2.0_YFP-channel.tif]
